# Fluoroquinolone resistance in *Salmonella*: insights by whole-genome sequencing

**DOI:** 10.1099/mgen.0.000195

**Published:** 2018-07-05

**Authors:** Wim L. Cuypers, Jan Jacobs, Vanessa Wong, Elizabeth J. Klemm, Stijn Deborggraeve, Sandra Van Puyvelde

**Affiliations:** ^1^​ Department of Biomedical Sciences, Institute of Tropical Medicine, Antwerpen, Belgium; ^2^​ Department of Mathematics and Computer Science, University of Antwerp, Antwerpen, Belgium; ^3^​ Department of Clinical Sciences, Institute of Tropical Medicine, Antwerpen, Belgium; ^4^​ Department of Microbiology and Immunology, Katholieke Universiteit Leuven (KU Leuven), Leuven, Belgium; ^5^​ Department of Medicine, University of Cambridge, Addenbrooke's Hospital, Cambridge, UK; ^6^​ Wellcome Trust Sanger Institute, Hinxton, UK

**Keywords:** *Salmonella*, fluoroquinolones, ciprofloxacin, antibiotic resistance, whole-genome sequencing

## Abstract

Fluoroquinolone (FQ)-resistant *Salmonella* spp. were listed by the WHO in 2017 as priority pathogens for which new antibiotics were urgently needed. The overall global burden of *Salmonella* infections is high, but differs per region. Whereas typhoid fever is most prevalent in South and South-East Asia, non-typhoidal salmonellosis is prevalent across the globe and associated with a mild gastroenteritis. By contrast, invasive non-typhoidal *Salmonella* cause bloodstream infections associated with high mortality, particularly in sub-Saharan Africa. Most *Salmonella* strains from clinical sources are resistant to first-line antibiotics, with FQs now being the antibiotic of choice for treatment of invasive *Salmonella* infections. However, FQ resistance is increasingly being reported in *Salmonella*, and multiple molecular mechanisms are already described. Whole-genome sequencing (WGS) is becoming more frequently used to analyse bacterial genomes for antibiotic-resistance markers, and to understand the phylogeny of bacteria in relation to their antibiotic-resistance profiles. This mini-review provides an overview of FQ resistance in *Salmonella*, guided by WGS studies that demonstrate that WGS is a valuable tool for global surveillance.

## Data Summary

Supplementary material is available with the online version of this article.

**Impact Statement**

In 2016, the United Nations General Assembly underlined the threat of antibiotic resistance and committed to join forces to combat this threat. Antibiotic resistance could cause a predicted 10 million deaths and have detrimental economic effects by 2050 if no actions are taken (https://amr-review.org/sites/default/files/160518_Final paper_with cover.pdf). In 2017, the WHO published a priority list of antibiotic-resistant bacteria, to support research and development of new antibiotics. Fluoroquinolone (FQ)-resistant *Salmonella* spp. were listed as a high priority. FQs have broad-spectrum activity and good pharmacokinetics for clinical use, and are important antibiotics for treatment of invasive bacterial infections, such as typhoid fever and invasive non-typhoidal *Salmonella* (iNTS) infections. Enteric fever (caused by the *Salmonella enterica* subspecies *enterica* serotypes Typhi and Paratyphi A, B and C) and iNTS (mainly caused by the serotypes Typhimurium and Enteritidis) have the highest impact and mortality in low- and middle-income countries. However, the resistance of *Salmonella* against FQs has been increasingly reported. The understanding of the FQ-resistance mechanisms and spread in *Salmonella* has significantly advanced through the implementation of whole-genome sequencing (WGS) during the past 5 years. Here, we review the genetic mechanisms of FQ resistance reported by WGS studies on *Salmonella*.

## Introduction

*Salmonellae* are Gram-negative bacteria, and strains that are pathogenic to humans are traditionally subdivided into two major groups based on their clinical presentation: typhoidal *Salmonella* and non-typhoidal *Salmonella* (NTS). Typhoidal *Salmonella,* comprising the *Salmonella enterica* subspecies *enterica* (hereafter *Salmonella*) serovars Typhi and Paratyphi A, B and C, cause a systemic disease, also known as enteric fever [1, 2]. Human-restricted *Salmonella* Typhi is the dominant cause of typhoid fever, with an estimated number of cases between 21.7 million [3] and 26.9 million per year [4], and an estimated 217 000 deaths per year [3]. The diverse group of NTS strains consists of more than 2500 serovars, which generally have different animals as hosts, and cause milder gastro-intestinal infections in humans, resulting in an estimated 93.8 million cases and 155 000 deaths each year [5]. However, some NTS strains, referred to as invasive NTS (iNTS), cause bloodstream infections with invasion of other organs. The global yearly burden of iNTS is estimated at 3.4 million infections and 681 316 deaths [6], and iNTS is highly prevalent in sub-Saharan Africa, where malnutrition, malaria and human immunodeficiency virus infections form major risk factors [7–9]. In sub-Saharan Africa, specific lineages of *Salmonella* serovars Typhimurium and Enteritidis have undergone genomic evolution associated with niche adaptation towards invasive disease in humans [10–13].

Multidrug resistance (MDR) in *Salmonella* is defined as co-resistance to the first-line antibiotics ampicillin, chloramphenicol and trimethoprim/sulfamethoxazole. The high prevalence of MDR in typhoidal *Salmonella* and iNTS necessitates the use of second-line antibiotics [14]. The fluoroquinolone (FQ) ciprofloxacin and the third-generation cephalosporin ceftriaxone are now the recommended drugs to treat invasive *Salmonella* infections or patients at risk of developing an invasive infection [15]. The macrolide antibiotic azithromycin can be used as an alternative [14]. Resistance to these recommended antibiotics is, however, increasingly described in *Salmonella* [9, 14, 16, 17]. The U.S. National Antimicrobial Resistance Monitoring System (NARMS) reported an increase in the percentage of *Salmonella* isolates that are non-susceptible [i.e. with minimum inhibitory concentration (MIC) values above the susceptibility breakpoint, see Supplementary Data S1, available with the online version of this article] to ciprofloxacin from <0.5 % up to 3.5 % since 1996 [18, 19]. Moreover, 6 % of *Salmonella* isolates were non-susceptible to ciprofloxacin in the EUCAST (European Committee on Antimicrobial Susceptibility Testing) database in 2015 [19].

In 2017, the WHO specifically ranked FQ resistant *Salmonella* as a high priority pathogen for the research and development of new antibiotics [20]. This ranking was based on ten criteria, of which FQ-resistant *Salmonellae* rank high for: (1) prevalence in the community, (2) transmissibility and zoonotic potential, (3) length of hospitalization after infection, and (4) unlikeliness of development of alternative antibiotics in the nearby future. Additional important criteria are the 10 year prevalence of FQ resistance among *Salmonella* Typhi and Paratyphi strains in the Americas, South Asia and South-East Asia, and the high mortality rates (up to 20 % associated with iNTS in sub-Saharan Africa [7, 20]). In this mini-review, we present and discuss the current situation of FQ resistance in *Salmonella*, guided by WGS studies, with a focus on molecular mechanisms.

## FQ: activity and resistance

Quinolones, such as nalidixic acid, are antibiotics that target the bacterial type II topoisomerases, and more specifically the DNA gyrase and the DNA topoisomerase IV [21]. Both proteins are encoded by the *gyrA*, *gyrB* and *parC*, *parE* genes, respectively, and modulate DNA supercoiling. Quinolones inhibit these enzymes, resulting in disrupted chromosome replication and rapid bacterial death [22–24]. FQs are quinolones with a single fluorine substituent, which increases DNA gyrase inhibitory activity and facilitates penetration into the bacterial cell [25–27]. While levofloxacin, gatifloxacin, moxifloxacin and gemifloxacin show the highest efficacy against Gram-positive bacteria, ciprofloxacin is most effective against Gram-negative bacteria, such as *Salmonella* [25].

Multiple resistance mechanisms against quinolones have been described in bacteria. First, mutations in the quinolone-resistance-determining regions (QRDRs) of the chromosomal *gyr* and *par* genes result in a lower quinolone-binding affinity of the topoisomerase enzymes [21, 28, 29]. Secondly, plasmid-mediated quinolone resistance (PMQR) involves acquisition of (i) *qnr* genes (*qnrA*, *qnrB*, *qnrS*, *qnrC*, *qnrD*), encoding topoisomerase-binding proteins that provide physical protection from quinolones [22, 30–32], (ii) the *aac(6′)-lb-cr* gene, encoding a modifying enzyme that decreases FQ activity [21, 23], and (iii) *oqxAB* and *qepA*, encoding quinolone efflux pumps [21, 25]. Finally, downregulation and upregulation of chromosome-encoded porins or multidrug efflux pumps (e.g. AcrAB-TolC), respectively, lower the cellular FQ concentrations [21, 22, 25].

Resistance against FQs is determined phenotypically, and the reference method uses measurement of the MIC for ciprofloxacin. Standardized cut-off values are provided by the Clinical and Laboratory Standards Institute (CLSI) and EUCAST. Resistance is defined as ciprofloxacin MIC values ≥1 µg ml^−1^, while MIC values ≤0.06 µg ml^−1^ indicate susceptibility [33]. Intermediate values are associated with treatment failure in *Salmonella* [34, 35], and are referred to as decreased ciprofloxacin susceptibility (DCS). A practical introduction to *in vitro* FQ susceptibility testing in *Salmonella* is provided in Supplementary Data S1. Detailed information on the definitions, molecular mechanisms and clinical impact of FQ susceptibility, DCS and FQ resistance is presented in Table S1. In this mini-review, we use the term ‘FQ resistance markers’ to group all molecular mechanisms that cause resistance to quinolones and non-susceptibility to FQs.

## FQ resistance in typhoidal *Salmonella*

**Fig. 1.**
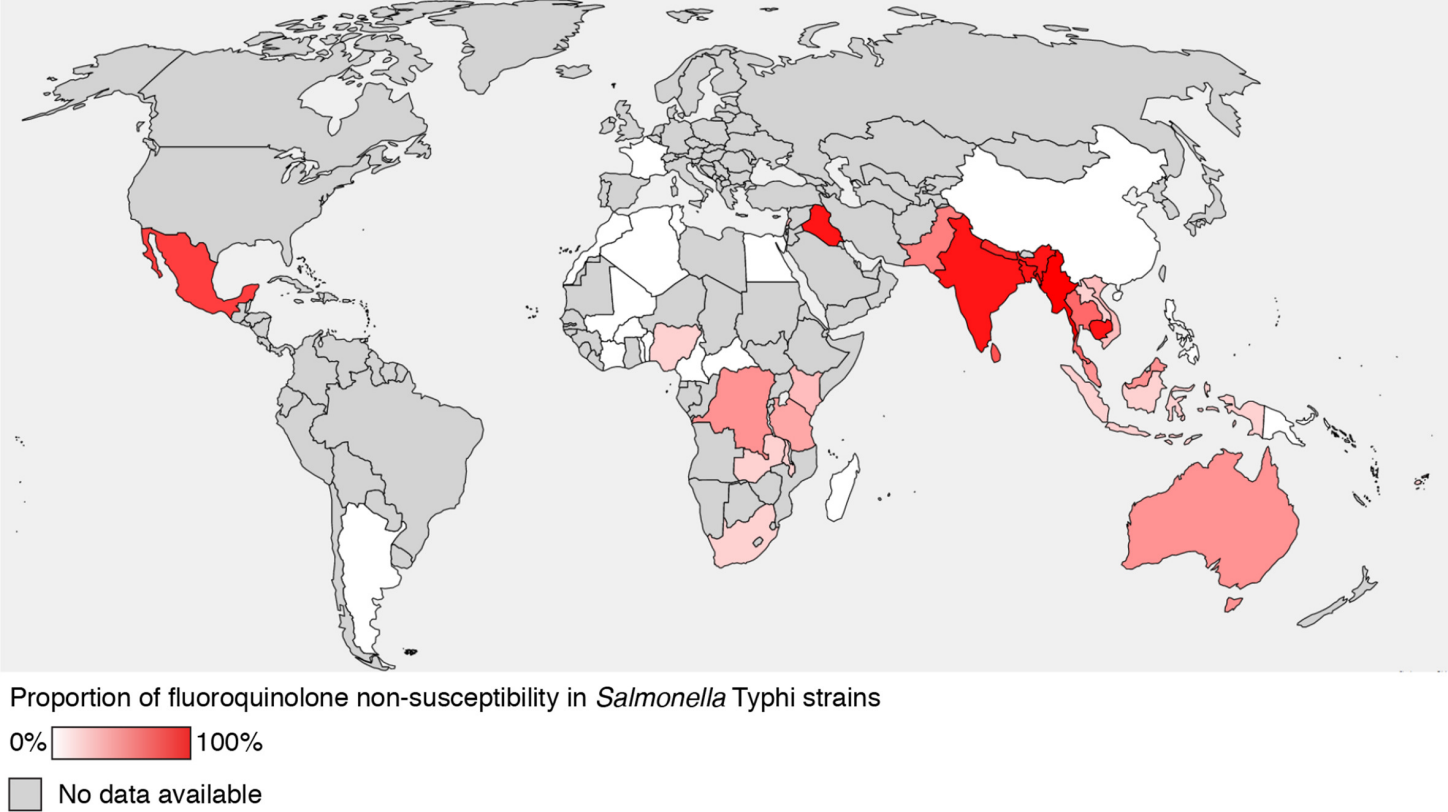
Percentage of FQ-resistance markers identified in whole-genome sequenced *Salmonella* Typhi isolates per country. The percentage of isolates carrying resistance markers are indicated with a colour gradient from 0 % (white) to 100 % (dark red). Countries for which no sequencing data is available are marked in grey. Data originates from the following studies: Wong *et al.* [36]; International Typhoid Consortium 2016 [43]; Hendriksen *et al.* [42]; Pham Thanh *et al.* [37]; Kuijpers *et al.* [39].

The implementation of WGS opened out our understanding of the prevalence and spread of FQ resistance in *Salmonella* Typhi. FQ resistance mechanisms in *Salmonella* Typhi as reported by WGS are summarized in Table 1 and the global distribution of FQ resistance in *Salmonella* Typhi is shown on the map in Fig. 1. In 2015, a large collaborative effort using WGS on 1832 isolates from 63 countries unravelled the global population structure of *Salmonella* Typhi [36]. The authors reported the spread of the dominant multidrug-resistant *Salmonella* Typhi clade H58 from Asia to East Africa and Oceania, which is more significantly associated with QRDR mutations (predominantly Ser83Phe, i.e. a point mutation in codon 83, resulting in a serine to phenylalanine amino acid change) compared to other *Salmonella* Typhi [36]. Multiple subsequent studies using WGS have reported FQ resistance markers in additional *Salmonella* Typhi isolates [36–40] (Table 1). Interestingly, accumulating mutations in the QRDR caused *Salmonella* Typhi to incrementally evolve towards increasing MIC values. Ciprofloxacin-susceptible strains (MIC≤0.06 µg ml^−1^) acquired a *gyrA* Ser83Phe single mutation causing DCS (MIC=0.12–0.5 µg ml^−1^) and additional *gyrA* and *parC* mutations, encoding Asp87Asn and Ser80Ile, respectively, caused high-level FQ resistance (MIC≥4 µg ml^−1^) [40]. Strains with multiple *gyr* and *par* mutations were reported from Cambodia, India and Nepal [36–39] (Table 1). Additionally, the *in vitro* evidence that QRDR mutations increase the fitness of *Salmonella* Typhi [41] is indicative that FQ resistance is irreversible and likely to remain.

**Table 1.** FQ-resistance markers in typhoidal salmonellae, reported by WGS *n* is the number of isolates sequenced, with superscript letters indicating whether the isolates were serotype Typhi (T) or Paratyphi A (P). The ‘% H58’ column indicates the percentage of Typhi isolates that were identified as part of the H58 clade for each region. The percentage of sequenced isolates containing FQ-resistance markers is reported under ‘% FQ^R^ markers’. The right panel of the table provides an overview of the identified FQ-resistance mechanisms per study. Each line represents a combination of FQ markers that was observed in the respective study. Mutations in gyrase (*gyr*) and topoisomerase IV (*par*) encoding genes are provided as resulting changes in residue, and presented per gene and per identified combination. na, Not available.

Reference	Region or country	*n*^T or P^	% H58	% FQ^R^ markers	FQ–resistance marker				
					PMQR	Mutations in *gyr* and *par*			
						*gyrA*	*gyrB*	*parC*	*parE*
[36]*	63 countries (Africa; Asia)	1832 (371; 1061)	47 (46; 62)	34 (10; 49)	–	Ser83Phe	–	–	–
					–	Ser83Phe	–	–	Asp420Asn
					–	Asp87Tyr	–	–	–
					–	–	Ser464Phe	–	–
					–	Ser83Tyr+Ser83Phe	–	Ser80Ile	–
					*qnrS*	–	–	–	–
[39]	Cambodia	64^T^	98	97	–	Ser83Phe	–	–	–
					–	–	Ser464Phe	–	–
					–	Ser83Phe+Asp87Asn	–	–	–
		21^P^	0	100	–	Ser83Phe	–	–	–
					–	Asp87Gly	–	–	–
[40]†	South Asia and South-East Asia	107^T^	73	na	–	Ser83Phe	–	–	–
					–	Ser83Tyr	–	–	–
					–	Asp87Asn	–	–	–
					–	Ser83Phe	–	Glu84Gly	–
					–	Ser83Phe	–	–	Asp420Asn
					–	Ser83Phe	Asp87Asn	Ser80Ile	–
[37]	Nepal	78^T^	83	81	–	Ser83Phe	–	–	–
					–	Ser83Phe+Asp87Asn	–	Ser80Ile	–
					–	Ser83Phe+Asp87Val	–	Ser80Ile	Ala364Val
[38]	Cambodia	209^T^	97	95	–	Ser83Phe	–	–	–
[43]	Nigeria	128^T^	0	5	*qnrS*	–	–	–	–
					–	Ser83Phe	–	–	–
					–	Ser83Tyr	–	–	–
[42]†	Zambia	32^T^	100	4	–	Ser83Tyr	–	–	–
					–	Asp87Asn	–	–	–
[46]†	DR Congo	1^T^	0	100	–	Ser83Phe	–	–	–
[47]†	India, New Delhi	4^T^	na	75	*qnrB*	–	–	–	–
[49]†	Pakistan	87^T^	100	100	*qnrS*	Ser83Phe	–	–	–

*Detailed information is provided at: www.stoptyphoid.org.

†Isolates were selected for their resistance properties prior to sequencing, i.e. implicates biased sampling.

In Africa, FQ-resistance markers were present in *Salmonella* Typhi H58 from Kenya, Tanzania, Malawi South Africa and Zambia [36, 42]. Interestingly, QRDR mutations were also reported in non-H58 Typhi in the Democratic Republic of the Congo (DR Congo) [36] and Nigeria [43] (Table 1). These studies suggest a lower prevalence and spread of FQ-resistance markers in Africa compared to Asia (Table 1, Fig. 1). Also in Africa, the *gyrA* Ser83Phe mutation was most frequently observed [44]. This may in part reflect the adaptability of *Salmonella* Typhi to changing antibiotic pressures with less FQ being used in Africa compared to Asia. However, given the varying incidence of typhoid fever between African regions [45] and the unavailability of bloodstream infection surveillance in large parts of Africa, the exact proportion of FQ-resistant strains in Africa remains elusive. For example, recently a single *Salmonella* Typhi isolate showing a Ser83Phe mutation in *gyrA* causing DCS, in combination with extended-spectrum β-lactamase (ESBL) production, was identified in the DR Congo [46]; in remote areas (such as in this report), it remains unclear whether such an isolate is part of a larger undetected outbreak with increased resistance.

Overall, PQMR in *Salmonella* Typhi is more rare than QRDR mutations and has been identified using WGS in isolates from Bangladesh [*qnrS1* on IncFIB(K) plasmid, *n*=5], South Africa [*qnrS2* on IncFIB(K) plasmid, *n*=1], India (*qnrB7* on IncX3 plasmid, *n*=4) and Nigeria (*qnrS* on Kpn3 plasmid, *n*=1) [36, 43, 47] (Table 1). This low prevalence of PMQR is in line with a recent meta-analysis of FQ-resistant *Salmonella* in Africa [44], and reports from Asia [48]. However, an ongoing outbreak of extensively drug-resistant and ESBL-producing *Salmonella* Typhi H58 from Pakistan was associated with QRDR mutations and the *qnrS* gene [49]. The presence of PMQR can provide a favourable environment for the selection of chromosomal QRDR mutations in *Salmonella* [19], which was also observed for other Enterobacteriaceae [50, 51].

Less WGS data are available for *Salmonella* Paratyphi A. In Cambodia, a recent increase of DCS in *Salmonella* Paratyphi A was predominantly associated with a Ser83Phe mutation in *gyrA* [39]. This is of significant interest, since *Salmonella* Paratyphi A infection is advancing in Asia [16, 17], while increasing DCS has been observed using conventional microbiological methods [52–55].

## FQ resistance in NTS

Foodborne infections with NTS are especially well documented in Europe and the USA, where frequencies of DCS and FQ resistance vary per serovar and country or region [56, 57]. Resistance at the human–animal interface is especially important for NTS, which have both animals and humans as potential hosts. Potential transmission of resistance is exemplified by recent findings that the resistance of *Salmonella* Typhimurium against ampicillin in the 1960s was related to the use of penicillin in animal feed in the late 1950s [58, 59]. Nowadays, FQs are extensively used in agriculture, and they additionally show a relatively low biodegradability [60]. FQs are still extensively used for animal production in several countries, e.g. for disease prevention and treatment in poultry [61]. Moreover, banning the use of FQs in food animals in Australia correlated with reduced FQ resistance in bacteria isolated from food, food animals and patients [62, 63].

PMQR can play an important role in spreading FQ resistance among strains at the human–animal interface. This is reflected by the higher numbers of the PMQR genes *qnr* and *oqx* detected by WGS studies in NTS (Table 2) compared to *Salmonella* Typhi (Table 1). In 2017, an integrated surveillance by several European reference laboratories allowed the linkage of an outbreak of *Salmonella* Chester to a food chain in Morocco [64]. One epidemic clone contained almost exclusively (87 %, *n*=96) isolates with PMQR markers [64] (Table 2). Toro *et al*. reported two *Salmonella* Enteritidis isolates from poultry in Chile carrying the *qnrB* gene [65]. One of the top five *Salmonella* serovars detected in humans in the USA is monophasic *Salmonella* Typhimurium, serotype 4,[4],12:i:- [56]. A recent WGS study (*n*=659) identified PMQR determinants in isolates from one multidrug-resistant clade of *Salmonella* serotype 4,[4],12:i:- originating from swine (Table 2), and the authors highlighted the risk as a potential reservoir for human infections [66].

**Table 2.** FQ-resistance markers in NTS, reported by WGS The number of isolates sequenced is indicated by ‘*n*’. ‘Source’ indicates whether samples were of human (H) or animal (A) origin. The percentage of sequenced isolates containing FQ-resistance markers is reported under ‘% FQ^R^ markers’. The right panel of the table provides an overview of the identified (combinations of) FQ-resistance mechanisms. Each line represents a combination of FQ markers that was observed in the respective study. Mutations in gyrase (*gyr*) and topoisomerase IV (*par*) encoding genes are provided as resulting changes in residue, and presented per gene and per identified combination. na, Not available.

Reference	Region or country	*n*	NTS serovar	Source	% FQ^R^ markers	FQ-resistance marker				
						PMQR	Mutations in *gyr* and *par*			
							*gyrA*	*gyrB*	*parC*	*parE*
[67]	Scotland	290	Typhimurium DT104	H, A	13	–	Ser83Phe	–	–	–
						–	Asp87G	–	–	–
						–	Asp87Asn	–	–	–
[10]	Africa, Asia, Europe, Americas	675	Enteritidis	H, A	0.15	–	–	–	–	–
	Africa	496				*qnrS*	–	–	–	–
[69]	USA	640	12 NTS serotypes	H	3	–	Asp87Tyr	–	–	–
						–	Ser83Phe	–	–	–
						–	Asp87Tyr+Ser83Phe	–	Ser80Ile	–
						*qnrS*	–	–	–	–
						*qnrB*	–	–	–	–
						*qnrB+oqxA+oqXB*	–	–	–	–
[68]	USA (New York and Washington)	90	Typhimurium	H	7	*qnrS*	–	–	–	–
						*oqxA*, *oqxB*	Asp87Tyr	–	–	–
						*oqxA*, *oqxB*	Ser83Tyr	–	–	–
						*qnrB*	–	–	–	–
						*oqxA*, *oqxB*	Asp87Asn	–	–	–
						–	Asp87Asn	–	–	–
[66]	USA, Europe	659	4,[4],12:i:-	A	5	*qnrB*, *qnrS*	–	–	–	–
[64]	Morocco, unknown	153	Chester	H	54	*qnrS*, *qnrB*	–	–	–	–
[81]	South Asia, South-East Asia and Oceania	115	Weltevreden	H, A	na	*qnrD*, *qnrS*	–	–	–	–
						*oqxA*, *oqxB*	–	–	–	–
[80]	Southern China	44	Weltevreden	H	5	*qnrD*	–	–	–	–
						*qnrS*	–	–	–	–
[65]	Chile	30	Enteritidis	A	7	*qnrB*	–	–	–	–
2018*	Vietnam	na	Typhimurium	H	na	*qnrS*	Asp87Asn	–	–	–

*S. Baker, personal communication (2018).

In contrast, a retrospective study from Scotland stated little evidence of *Salmonella* Typhimurium DT104 transmission between human and animal reservoirs; some strains also contained FQ-resistance markers [67] (Table 2). Similar results were reported for *Salmonella* Typhimurium in the USA, in which strains isolated from humans contained a more diverse repertoire of resistance markers, including QRDR mutations (Table 2), compared to bovine isolates [68]. In a WGS study from the USA on NTS isolated from retail meat and human patients, only strains isolated from humans contained FQ-resistance markers [69]. WGS allows the study of transmission events with an unprecedented resolution, but interdisciplinary and inter-sectorial research will be required to fully elucidate and monitor the drivers of resistance in NTS.

In lower-income and middle-income countries, iNTS infection is highly prevalent and associated with high mortality [8]. For invasive *Salmonella* Enteritidis in Africa, the prevalence of FQ-resistance markers is low (Table 2). Among 496 *Salmonella* Enteritidis isolates originating from African countries, only 1 isolate had a *qnrS* gene [10] (Table 2). Large studies focussing on *Salmonella* Typhimurium and other NTS serotypes are limited, and only a few have applied WGS. Although FQ-resistance levels are low in most studies in Africa [70, 71], several small-scale studies report FQ-resistance markers in specific areas, ranging from mutations conferring DCS [44, 70, 72–77], up to high-level FQ resistance conferred by two *gyrA* mutations (Ser83Phe and Asp87Gly), a *parC* (Ser80Ile) mutation and an additional PMQR gene [*aac(6′)-Ib-cr*] [78]. In Asia, the burden of iNTS is much lower than the burden of typhoid fever [79]. PQMR genes have been reported in isolates of *Salmonella* Weltevreden from Asia (Table 2), a serotype that can potentially cause invasive infections [80, 81]. In Vietnam, WGS revealed a new clone of invasive *Salmonella* Typhimurium, which is associated with human immunodeficiency virus patients, and some isolates showing QRDR mutations and PMQR (S. Baker, personal communication) (Table 2).

## Conclusions

FQ resistance in *Salmonella* seriously compromises treatment options, especially for invasive salmonellosis. The dominant presence of the *Salmonella* Typhi H58 clade associated with QRDR mutations jeopardizes effective FQ treatment of typhoid fever in Asia. Recent reports from Nepal indicated that even the fourth-generation FQ gatifloxacin has lost its effectiveness due to high-level FQ resistance [52, 82]. WGS data on FQ-resistant iNTS are rare and this can be due to the low resistance levels reported in most studies in Africa, while the burden of iNTS is the highest in this region. Because FQ resistance may be emerging [70], large multi-country studies are required to monitor the presence and spread of FQ resistance in iNTS in Africa. For NTS, both animals and humans are potential hosts, and from the existing literature, it is clear that there is a higher diversity of PMQR mechanisms in NTS compared to typhoidal *Salmonella*. This might be linked to a diverse host niche, including several animal reservoirs, indicative of the need for a ‘one health’ approach to efficiently monitor the spread and source of FQ resistance.

The increasing use of WGS provides new molecular surveillance approaches to monitor and understand the spread of FQ resistance in *Salmonella*. Whereas originally predominantly used for research, WGS is becoming more available in diagnostic laboratories across the world and tools are being developed to facilitate the data analyses (such as www.WGSA.net).

In summary, FQ resistance in *Salmonella* spp. is rising towards critical levels and there is need for alternatives, such as last resort antibiotics and the development of new antibiotics, as stated by the WHO in 2017 [20]. Further monitoring will be critical in the coming years to analyse the evolution of *Salmonella* strains and their resistance patterns. Hereto, the implementation of WGS provides new opportunities for surveillance.

## Supplementary Data

Supplementary File 1
