# Supplementary material for: Fluoroquinolone resistance in Salmonella: insights by whole-genome sequencing
Source: Microb Genom. 2018 Jul 5;4(7):e000195. doi: 10.1099/mgen.0.000195 (PMC6113872; doi:10.1099/mgen.0.000195)
Supplement: Supplementary File 1 [file mgen-4-195-s001.pdf]

Supplemental Data S1

Box S1: Fluoroquinolone susceptibility testing in the diagnostic laboratory

There are several methods to perform fluoroquinolone susceptibility testing of *Salmonella*:

- **Disk diffusion generating inhibition zone diameters (in mm):** antibiotic-containing disks are added to a culture of the bacteria inoculated on solid agar medium (Fig. SB1). In case of susceptibility, there will be no growth in an “inhibition zone” around the disk (the diameter is measured and rounded to the nearest whole mm). The larger the inhibition zone, the more active the antibiotic is towards the bacterium. Disk diffusion is unreliable for testing *Salmonella* fluoroquinolone susceptibility.
- **Broth microdilution testing generating minimal inhibitory concentrations (MICs) in (µg/mL):** antibiotics are added in serial dilutions to tubes or wells of bacteria grown in liquid culture (broth). The MIC value is defined as the lowest concentration of antibiotic at which the bacteria are inhibited (*i.e.* do not show growth) and is expressed as µg/mL (or mg/L). The lower the MIC value, the more active is the antibiotic. Currently, CLSI* and EUCAST* recommend MIC-testing to predict susceptibility of *Salmonella* to fluoroquinolones
- **Ciprofloxacin Etest:** if broth microdilution is not possible, the Etest is alternative. The Etest consists of an antibiotic-impregnated strip with a gradient of antibiotic concentration (Fig. SB2), and allows determining MIC values in good agreement with microbroth testing [1].
- **Surrogate disk diffusion test:** the results of pefloxacin disk diffusion testing are predicting susceptibility and resistance of *Salmonella* to ciprofloxacin [2] and is therefore an alternative for low-resource settings. Pefloxacin disk diffusion testing is however not indicative for the *aac(6')-Ib-cr* PMQR mechanism [2]. Prior to pefloxacin, nalidixic acid (a nonfluorinated quinolone) disk diffusion was used as a predictor for ciprofloxacin susceptibility testing, but its susceptibility was not affected by *gyrB* and PMQR mechanisms.

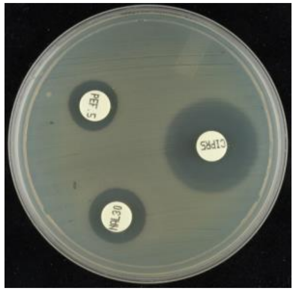

Fig. SB1: Disk diffusion with *Salmonella* Typhi susceptible to ciprofloxacin, resistant to pefloxacin and resistant to nalidixic acid

Zone diameters and MICs are interpreted based on ‘cut-off values’ or ‘breakpoints’. The following interpretative categories can be distinguished. The breakpoints for fluoroquinolone activity against *Salmonella* are given in Table SB1 and Table SB2.

- Bacteria with a MIC at or below (or with a zone diameter at or above) the susceptibility breakpoint are categorized as **Susceptible** (S): at the recommended dosage, clinical efficacy is predicted.
- Bacteria with a MIC at or above (or with a zone diameter at or below) the resistance breakpoint are categorized as **Resistant** (R): at the recommended dosage, clinical efficacy is not assured.
- Bacteria with a MIC or a zone diameter in between the susceptible and resistance breakpoints are categorized as **Intermediate** (I): this category implies clinical efficacy in body sites where antibiotics are physiologically concentrated (*e.g.* urine) or when a higher than normal dosage of an antibiotic can safely be used. For invasive *Salmonella* infections, this clinical use and category are not applicable.

*Note: Guidance to quality-assured antimicrobial susceptibility testing is provided by (supra)national institutes such as the Clinical and Laboratory Standards Institute (CLSI) and The European Committee on Antimicrobial Susceptibility Testing (EUCAST).

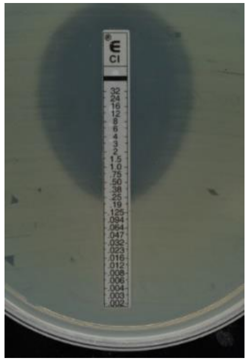

Fig. SB2: E-test with *Salmonella* Typhi: MIC-value of 0.25 µg/ml

Table SB1: Cut-off values of zone diameters for disk diffusion testing for invasive *Salmonella* according to the CLSI M100-S28 and EUCAST Clinical Breakpoint Table v. 8.0, 2018.

Antibiotic (5µg)	CLSI Interpretative Categories and zone diameter breakpoints (mm)			EUCAST zone diameter breakpoints (mm)	
	S	I	R	S	R
Ciprofloxacin	≥ 31	21 -30	≤ 20	-	-
Pefloxacin	≥ 24	-	≤ 23	≥ 24	< 24

Table SB2: MIC breakpoints for invasive *Salmonella* according to the CLSI M100-S28 and EUCAST Clinical Breakpoint Table v. 8.0, 2018

Antibiotic	CLSI Interpretative Categories and MIC breakpoints (µg/mL)			EUCAST MIC breakpoints (µg/mL)	
	S	I	R	S	R
Ciprofloxacin	≤ 0.06	0.12 – 0.5	≥ 1	≤ 0.06	> 0.06

Table S1: Categories of fluoroquinolone susceptibility and resistance of invasive *Salmonella*, in particular for *Salmonella* Typhi and Paratyphi. Categories are ranked according to increasing resistance. Definitions, molecular mechanisms, clinical impact, and occurrence of the resistance are described, as well as the successive adaptations of EUCAST and CLSI guiding documents. Abbreviations: QRDR = quinolone resistance-determining region, PMQR = plasmid-mediated quinolone resistance.

	**Ciprofloxacin susceptible**	**DCS** [3] **or Low-level FQ Resistance** [4]**, or Reduced FQ Susceptibility** [5] **or Intermediate Susceptible** [6]	**High-level FQ Resistance** [3] **or Full clinical resistance to ciprofloxacin** [7]
**Definition**	ciprofloxacin MIC ≤ 0.064 µg/mL [6][8].	ciprofloxacin MIC > 0.064µg/mL and < 1 µg/mL	ciprofloxacin MIC value ≥ 4µg/ml (referring to the CLSI 2011 version) [3]<br>"Full clinical resistance" was used to define a ciprofloxacin MIC ≥ 2µg/ml [7], *i.e.* above the actual newer resistance breakpoint of the CLSI guidelines since 2013 [9].
**Molecular mechanisms**	No mutations in the QRDR regions of the *gyrA* and *parC* genes [10].	Single chromosomal point mutations in the QRDR regions of the *gyrA*, *gyrB*, *parC* or *par*E genes [3]<br>PMQR genes (*qnr* variants and *aac(6')-Ib-cr*)	Two or more mutations in the *gyrA* gene, and an additional *par* mutation [3, 10]<br>Efflux pump mechanisms can be involved [11]
**Clinical implications**	Treatment with FQ (ciprofloxacin) is predicted to be successful	The fourth-generation gatifloxacin remained efficacious for treatment of non-complicated infections caused by DCS *Salmonella* Typhi strains [12, 13].	*Salmonella* Typhi isolates, with ciprofloxacin MIC values ≥ 16 µg/ml and gatifloxacin MIC values ≥ 1 µg/mL were associated with therapeutic failure of gatifloxacin during a clinical trial in Nepal [14].
**Occurrence of resistance**		*Salmonella* Typhi DCS strains are now worldwide dominating, partly catalyzed by the spread of the H58 clade [15]	Until recently, full resistance to ciprofloxacin was rare; it was mainly confined to *Salmonella* Typhi/Paratyphi A in India and Tajikistan [7].

References

1. **Deak E, Skov R, Hindler JA, Humphries RM**. Evaluation of surrogate disk tests for detection of ciprofloxacin and levofloxacin resistance in clinical isolates of *Salmonella* enterica. *J Clin Microbiol* 2015;53:3405–3410.
2. **Fang FC**. Fluoroquinolone Resistance in *Salmonella* and the Utility of Perfloxacin Disk Diffusion. *J Clin Microbiol* 2015;53:3401–3405.
3. **Humphries RM, Fang FC, Aarestrup FM, Hindler JA**. In vitro susceptibility testing of fluoroquinolone activity against *Salmonella*: Recent changes to CLSI standards. *Clin Infect Dis* 2012;55:1107–1113.
4. **European Committee on Antimicrobial Susceptibility Testing**. Breakpoint tables for interpretation of MICs and zone diameters. Version 1.0. http://www.eucast.org/ast_of_bacteria/previous_versions_of_documents/ (2009).
5. **Clinical and Laboratory Standards Istitute**. *Performance Standards for Antimicrobial Susceptibility Testing; Twenty-First Informational Supplement*. CLSI document M100-S21. Wayne, PA: Clinical and Laboratory Standards Institute; 2011.
6. **Clinical and Laboratory Standards Institute**. *Performance Standards for Antimicrobial Susceptibility Testing.* 28th ed. CLSI supplement M100. Wayne, PA: Clinical and Laboratory Standards Institute; 2018.
7. **Crump JA, Sjölund-Karlsson M, Gordon MA, Parry CM**. Epidemiology, clinical presentation, laboratory diagnosis, antimicrobial resistance, and antimicrobial management of invasive *Salmonella* infections. *Clin Microbiol Rev* 2015;28:901–937.
8. **European Committee on Antimicrobial Susceptibility Testing**. Breakpoint tables for interpretation of MICs and zone diameters. Version 8.0. http://www.eucast.org (2018).
9. **Clinical and Laboratory Standards Institute**. *Performance Standards for Antimicrobial Susceptibility Testing; Twenty-Third Informational Supplement*. CLSI document M100-S23. Wayne, PA: Clinical and Laboratory Standards Institute; 2013.
10. **Matono T, Morita M, Yahara K, Lee K, Izumiya H, *et al.*** Emergence of Resistance Mutations in *Salmonella* enterica Serovar Typhi Against Fluoroquinolones. *Open Forum Infect Dis* 2017;4:1–17.
11. **Keddy KH, Smith AM, Sooka A, Ismail H, Oliver S**. Mechanisms of resistance in nontyphoidal *Salmonella* enterica strains exhibiting a nonclassical quinolone resistance phenotype. *Emerg Infectuous Dis* 2010;15:879–880.
12. **Koirala S, Basnyat B, Arjyal A, Shilpakar O, Shrestha K, *et al.*** Gatifloxacin Versus Ofloxacin for the Treatment of Uncomplicated Enteric Fever in Nepal: An Open-Label, Randomized, Controlled Trial. *PLoS Negl Trop Dis* 2013; 7:e2523.
13. **Arjyal A, Basnyat B, Koirala S, Karkey A, Dongol S, *et al.*** Gatifloxacin versus chloramphenicol for uncomplicated enteric fever: An open-label, randomised, controlled trial. *Lancet Infect Dis* 2011;11:445–454.
14. **Arjyal A, Basnyat B, Nhan HT, Koirala S, Giri A, *et al.*** Gatifloxacin versus ceftriaxone for uncomplicated enteric fever in Nepal: An open-label, two-

centre, randomised controlled trial. *Lancet Infect Dis* 2016;16:535–545.

15. **Wong VK, Baker S, Pickard DJ, Parkhill J, Page AJ, *et al.*** Phylogeographical analysis of the dominant multidrug-resistant H58 clade of *Salmonella* Typhi identifies inter-and intracontinental transmission events. *Nat Genet* 2015;47:632–639.
